# SFRP4 drives invasion in gastric cancer and is an early predictor of recurrence

**DOI:** 10.1007/s10120-020-01143-8

**Published:** 2020-12-04

**Authors:** Rita A. Busuttil, Joshy George, Colin M. House, Stephen Lade, Catherine Mitchell, Natasha S. Di Costanzo, Sharon Pattison, Yu-Kuan Huang, Patrick Tan, Jae-Ho Cheong, Sun Young Rha, Alex Boussioutas

**Affiliations:** 1 Upper Gastrointestinal Translational Research Laboratory, Peter MacCallum Cancer Centre, 305 Grattan St, Parkville, VIC Australia; 2 Sir Peter MacCallum Department of Oncology, The University of Melbourne, Parkville, VIC Australia; 3 Department of Medicine, The University of Melbourne, Parkville, VIC Australia; 4 Computational Sciences, Jackson Laboratory for Genomic Medicine, Farmington, USA; 5 Department of Pathology, Peter MacCallum Cancer Centre, East Melbourne, VIC Australia; 6 Department of Medicine, University of Otago, Dunedin, New Zealand; 7 Genome Institute of Singapore, Singapore, Singapore; 8 Cancer Science Institute of Singapore, Yong Loo Lin School of Medicine, National University of Singapore, Singapore, Singapore; 9 Department of Surgery, Yonsei University College of Medicine, Seoul, Republic of Korea; 10 Department of Biomedical Systems Informatics, Yonsei University College of Medicine, Seoul, Republic of Korea; 11 Division of Medical Oncology, Yonsei Cancer Center, Yonsei University College of Medicine, Seoul, Republic of Korea

**Keywords:** SFRP4, Gastric cancer, Invasion, Biomarker, Recurrence

## Abstract

**Objective:**

Gastric cancer patients generally have a poor outcome, particularly those with advanced-stage disease which is defined by the increased invasion of cancer locally and is associated with higher metastatic potential. This study aimed to identify genes that were functional in the most fundamental hallmark of cancer, namely invasion. We then wanted to assess their value as biomarkers of gastric cancer progression and recurrence.

**Design:**

Data from a cohort of patients profiled on cDNA expression arrays was interrogated using K-means analysis. This genomic approach classified the data based on patterns of gene expression allowing the identification of the genes most correlated with the invasion of GC. We evaluated the functional role of a key protein from this analysis in invasion and as a biomarker of recurrence after curative resection.

**Results:**

Expression of secreted frizzled-related protein 4 (SFRP4) was identified as directly proportional to gastric cancer invasion. This finding was validated in multiple, independent datasets and its functional role in invasion was also confirmed using invasion assays. A change in serum levels of SFRP4 after curative resection, when coupled with AJCC stage, can accurately predict the risk of disease recurrence after curative therapy in an assay we termed *PredictR*.

**Conclusions:**

This simple ELISA-based assay can help predict recurrence of disease after curative gastric cancer surgery irrespective of adjuvant therapy. The results require further evaluation in a prospective trial but would help in the rational prescription of cancer therapies and surveillance to prevent under or over treatment of patients after curative resection.

**Supplementary Information:**

The online version contains supplementary material available at 10.1007/s10120-020-01143-8.

## Introduction

Gastric cancer (GC) is the fifth most common cancer worldwide and the third-highest cause of cancer-related deaths [1]. In countries lacking established screening programs GC is often diagnosed at an advanced stage contributing to the high mortality rate. In most Western countries the overall 5 year survival rate is less than 30% [2].

Although there are a variety of prognostic measures [3, 4], the best predictor of recurrence for GC is the pathological TNM stage using the AJCC/UICC staging system. Invasion, measured by T-stage, is a fundamental property of cancer [5, 6] and the identification of key genes involved causally in invasion represent ideal candidates for therapeutics and diagnostic markers for GC.

We previously utilized mRNA expression analysis of tumour and adjacent normal samples of gastric origin to identify molecular signatures characteristic of premalignant lesions and of histological subtypes of GC [7]. The same data set has been interrogated in this study and has identified secreted frizzled-related protein 4 (*SFRP4*) as a gene highly correlated with tumour invasion or T-stage. *SFRP4*’s role in invasion was validated using independent gene expression data sets, immunohistochemistry and in vitro model systems. We then exploited the secreted nature of the SFRP4 protein to develop an ELISA-based assay (*SFRP4 ratio*) which, when used in conjunction with existing clinical variables, is able to predict disease recurrence in GC patients after curative resection with a high degree of accuracy.

## Materials and methods

### Patients and samples

Tumour samples were collected from patients undergoing curative resection for GC in Melbourne. Blood samples were collected at the time of surgery and at indicated intervals post-operatively.

### Microarray analysis

cDNA microarrays (GSE2669) on tumour specimens were run previously [7]. Clinical characteristics of patient samples are described in Table S1. All continuous variables were considered as parametric data and analysed with ANOVA using the Benjamini–Hochberg false discovery rate for multiple correction [8]. K-means clustering analysis did not include correction for multiple testing and was conducted in Genespring version 4.2 (Agilent Technologies Inc., California).

### Microarray Validation cohorts

Validation cohorts were selected based on their genomic platforms and the availability of clinical information. Australian Gastric (*n* = 99; GSE51105) [9] (see Table S1**)** and Singapore Gastric (*n* = 178; GSE15459)[10] cohorts were profiled using Affymetrix U133 plus2 arrays. The TCGA STAD gastric cohort was profiled using RNA-seq [11].

### Immunohistochemistry

Immunostaining was performed on tissue microarrary (TMA) sections using anti-SFRP4 antibodies (1:250; provided by Lisa Horvath [12]) and the DAKO LSAB + kit (DAKO), following the manufacturer’s protocol.

### Overall survival and relapse-free survival analysis

Australian and Singapore datasets were analysed for Relapse-free (RFS) and overall survival (OS) using the Barcode [13] algorithm. Validation using the public database Kaplan–Meier plotter (KMplot) [14] was performed using default settings. OS of the TCGA dataset was interrogated using the Survexpress portal [15].

### Genes and pathways correlated with SFRP4

Genes correlated with *SFRP4* were identified in the Australian and TCGA cohorts using R and the cBIOPORTAL [16, 17] interfaces respectively. Genes with Pearson correlation ≥ 0.6 were identified and duplicate genes were removed. Genes common to both datasets were analysed using Reactome to identify functional pathways [18].

### Cell culture

The human GC cell lines AGS, SNU-1 and NCI-N87 were obtained from the American Type Culture Collection (Rockville, MD, USA). Cells were cultured in DMEM (AGS) or RPMI (SNU-1 and NCI-N87) supplemented with 10% fetal bovine serum, penicillin (100 U/ml) and streptomycin (100ug/ml) (all from Invitrogen, Carlsbad, CA). Cell lines were verified mycoplasma negative and identities were verified using the PowerPlex HS16 System kit (Promega).

### shRNA knockdown

Verified shRNA clones were obtained from the Open Biosystems pGIPz library (Victorian Centre for Functional Genomics). shRNA-expressing lentiviral plasmids were transfected using Lenti-X packaging vectors into HEK293T cells (Open Biosystems). Viral containing media was collected, filtered and stored at  − 80 °C. Target cells were transduced and selected using puromycin. Knockdown was confirmed using quantitative real-time PCR and Western blot.

### Western blotting

Total protein was extracted using lysis buffer(50 mM Tris pH 8.0, 2% SDS). Western blot analysis was carried out using standard procedures with the following antibodies: anti-SFRP4 polyclonal antibody [R&D (AF1827) 1:10,000] and Anti-Tubulin (Sigma, clone B-5-1-2).

### Proliferation and apoptosis assays

Proliferation assays were performed by seeding 5 × 10^4^ cells/well in 6-well plates and cell numbers were determined daily using the Countess (Invitrogen) system for a total of 3 days. Apoptosis was detected using the Apoptaq kit (Chemicon) using the manufacturer’s conditions. AGS and NCI-N87 cells were seeded directly into chamber slides whilst a cytospin was performed on SNU-1 cells to adhere them to the slide prior to fixing in methanol.

### Invasion assay

Cells were synchronized by serum starvation to prevent proliferation during the assay. 400,000 cells in serum-free media ± recombinant SFRP4 (0–20 nM; R&D systems) were combined with matrigel and seeded into 8 µm pore transwells. Media containing serum was added to the bottom chamber as a chemoattractant and incubated for 24 h at 37 °C/5% CO_2_. Migrated cells were fixed in formalin and stained with DAPI. Membranes were mounted on slides and quantitated by microscopy. Five microscopic fields were counted per membrane. Each experiment was performed in triplicate at least three times.

### Wound healing (migration) assay

AGS cells were seeded in a dark-walled 96-well plate and cultured till confluent then serum starved for 24 h. The cell monolayer was wounded with a 1.5 × 4 mm scratch using a robotically driven pin and growth media replaced After 24 h cells were fixed using 2% PFA and stained with phalloidin (Molecular Probes) and DAPI. The extent of wound healing determined using published methods [19].

### SFRP4 ELISA

**Fig. 1:**
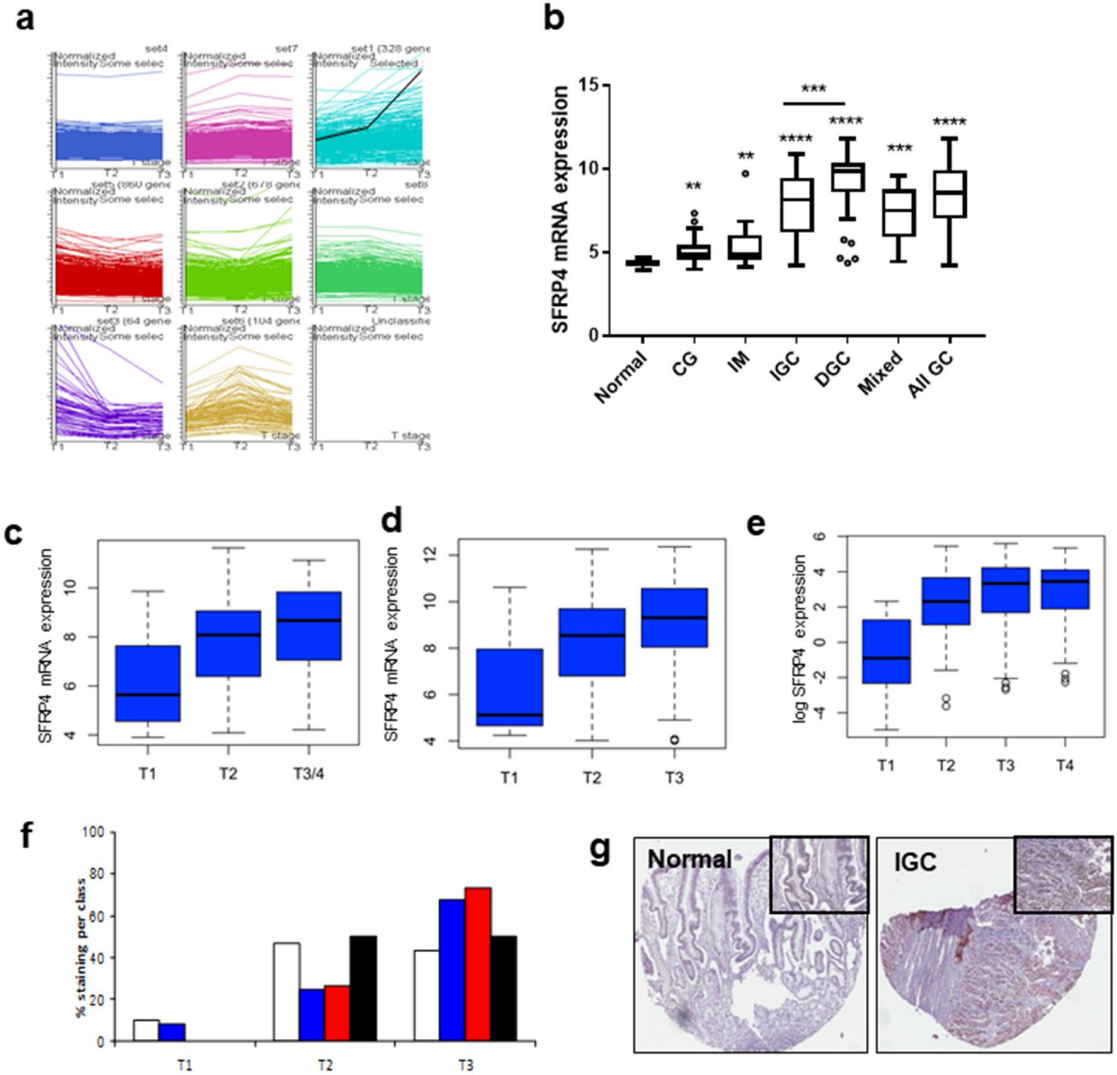
Identification of SFRP4 as the gene most correlated with invasion. cDNA array expression data was generated for 65 tumours of known T-stage. **a** K means clustering was performed based on depth of invasion (T stage). Overall patterns of gene expression (*y*-axis) using depth of invasion as a continuous variable (x-axis) visualising all 7383 genes were generated. SFRP4 (black line) was identified as the gene most correlated with invasion in this dataset. Findings were then validated over independent platforms and datasets. **b** SFRP4 expression in gastric tissues. 146 gastric tissues were profiled using Affymetrix U133 plus 2 arrays and stratified according to tissue type. Histologically normal gastric tissues [NN (*n* = 7)] exhibited significantly lower SFRP4 expression than other benign tissues [chronic gastritis (CG (*n* = 22); *p* = 0.0077)] and intestinal metaplasia [IM (*n* = 23; *p* = 0.0031)]. Highest levels of SFRP4 expression were observed in gastric tumour samples (*n* = 99; *p* < 0.0001). Further analysis of the 2 histological subtypes showed SFRP4 expression to be highest in diffuse GC (*n *= 39) compared to intestinal gastric cancer (*n* = 50; *p* = 0.0004). Mann–Whitney test was used for analysis. SFRP4 expression based on T-stage was determined in (**c**) an updated Australian data set of 99 tumours (*p* = 0.003; Kruskal–Wallis test) and the (**d**) Singapore dataset (*n* = 178; *p* = 0.009 Kruskal–Wallis test) were run on Affymetrix Human U133 plus 2 arrays. Each panel represents an individual probe for SFRP4 on the array. **e** The data was also validated using the TCGA RNASeq data set (*n* = 255; *p* = 2 × 10^–5^ Kruskal–Wallis test) (**f**) SFRP4 protein expression was determined by IHC on a TMA. Staining was quantitated using a semi-quantitative scale from 0 (no staining; white bar), 1 + (blue bar), 2 + (red bar) and 3 + (black bar) (**h**) representative images showing staining of normal gastric mucosa and an intestinal type GC (IGC). Images × 10 and magnified × 40

SFRP4 ELISA was performed using a commercially available kit (USCNK Life Science Inc) following the manufacturer’s instructions. Samples represent unique cohorts of pre- and post-operative specimens and with at least 3 years follow up. A consort diagram (FigS1) details the criteria for sample selection. An independent validation dataset was obtained from South Korea. Clinical characteristics for all cohorts are detailed in Table1.

**Table 1:** Clinical characteristics of ELISA cases

	Pilot set		Test set		Korean set	
Parameter	Recurrence	Non-recurrence	Recurrence	Non-recurrence	Recurrence	Non-recurrence
Age at surgery [years (range)]	60.7 (33–83)	67.1 (55–78)	63.6 (33–83)	64.13 (43–78)	53 (33–75)	60 (43–81)
Gender						
Male	8	10	28	21	3	17
Female	3	3	8	10	3	5
Type II diabetes						
Y	0	1	4	4	1	3
N	11	12	32	27	5	19
Tumour location						
Cardia	4	1	8	5	0	0
Non cardia	7	12	28	26	6	22
*H. pylori* status						
Positive	6	6	19	17	2	3
Negative	4	4	9	10	2	9
Unknown	1	3	8	4	2	10
Chemo-radiotherapy						
Neo-Adjuvant						
No Adjuvant	9	13	33	30	6	22
Adjuvant	2	0	3	1	0	0
Adjuvant						
No Adjuvant	4	7	12	20	UNK	UNK
Adjuvant	7	6	24	11	UNK	UNK
Palliative						
No Palliative	3	N/A	19	N/A	UNK	N/A
Palliative	8	N/A	13	N/A	UNK	N/A
ND	0	N/A	4	N/A	UNK	N/A
Pathology						
Diffuse	4	3	14	8	2	15
Intestinal	6	8	12	20	3	7
Mixed	1	0	7	1	1	0
Adenocarcinoma	0	1	2	1	0	0
Adenosquamous	0	1	1	1	0	0
Differentiation						
Well	0	1	0	1	0	0
Moderate	4	4	10	10	2	5
Poor	6	6	20	18	3	13
Undifferentiated	1	2	6	2	1	4
T stage						
T1	0	2	0	7	0	0
T2	3	4	5	11	0	1
T3	8	7	29	13	1	8
T4	0	0	2	0	5	13
AJCC 6th stage						
IA	0	2	0	7	0	0
IB	1	2	1	7	0	0
II	3	5	10	9	0	0
IIIA	5	2	16	4	0	4
IIIB	1	2	3	4	1	5
IV	1	0	6	0	5	13
Surgery type						
Proximal gastrectomy	3	1	6	1	0	0
Distal gastrectomy	4	6	11	14	0	15
Total gastrectomy	1	5	15	15	6	7
Oesophagogastrectomy	3	1	4	1	0	0
Margins						
R0	11	12	32	30	4	22
R1	0	1	4	1	2	0
Recurrence type						
Local	1	0	3	0	0	0
Distant	5	0	22	0	5	0
Both	5	0	11	0	1	0
None	0	13	0	31	0	22
Time till recurrence	25.6 (5.9-43.5)	N/A	21.1 (2.8-77.4)	N/A	93 (30.6-233.23)	N/A

*N/A* not applicable

*UNK* unknown

### Statistical analysis

Data are expressed as mean ± SEM unless otherwise indicated. Analysis was performed using Graphpad prism software. K-means clustering was performed using cDNA expression arrays using Genespring software (Agilent, USA). All analysis of Affymetrix U133 plus 2 data was carried out using R-package. We used SFRP4 ratio and TNM stage information as independent variables in a binary logistic regression analysis which resulted in a predicted probability of the combination of both variables predicting recurrence. This was evaluated by AROC and all analyses were performed in SPSS ver22 for Mac (IBM, Chicago).

## Results

### SFRP4 expression correlates with invasion

Invasion is a necessary requirement of cancer. To identify key genes contributing to invasion, cDNA expression array data previously generated in our laboratory using an Australian based cohort of 65 tumours was interrogated (GSE2669; See Table S1). T-stage was used as a measure of invasion of GC. Expression patterns positively correlated with T-stage were identified using the GeneSpring (Agilent Technologies Inc., California) K-means clustering algorithm. Eight patterns (K) of gene expression were chosen where every element on the 10.5 K array (incorporating 7383 genes) was used.

*SFRP4* has been previously identified by our group as significantly overexpressed in GC compared to normal tissue [7] and in this study was identified as one of the most significantly correlated genes with T-stage in this discovery cohort (Fig. 1a) and given its secreted nature was selected for further investigation.

A second Australian based cohort was utilised for further validation. This cohort consisted of 99 GC cases (including 43 samples which were also profiled in the cDNA array discovery cohort) and 40 normal/premalignant samples profiled using Affymetrix 133 plus2 arrays (GSE51105; Table S1).

The expression of *SFRP4* during progression to GC was explored(Fig. 1b). Compared to normal gastric mucosa (*n* = 7) the premalignant conditions chronic gastritis (*n* = 22, *p* = 0.0077) and intestinal metaplasia (*n* = 21, *p* = 0.0031) both demonstrated elevated *SFRP4* expression. *SFRP4* expression was highest in the all GC group which comprised all GC cases (*n* = 99; *p* < 0.0001 compared to normal). Further analysis of the GC cases showed the diffuse subtype (*n* = 39) had significantly higher SFRP4 expression than the intestinal subtype (*n* = 50; *p* = 0.0004).

Increasing SFRP4 expression with T-stage was validated in three independent GC cohorts. Analysis of the tumour samples in the Australian cohort (described above) showed that *SFRP4* expression levels increased incrementally with advanced T-stage (Fig. 1c; *p* = 0.003; Kruskal–Wallis test). Due to the limited number of T4 samples available, these were grouped with the T3 samples. Expression data were obtained from 178 independent GC samples originating from Singapore. T-stage information was available for 152 of these cases. *SFRP4* mRNA expression data for these cases is represented in Fig. 1d (*p* = 0.009; Kruskal–Wallis test). The STAD-TCGA dataset contains RNA-Seq data for 255 GC cases with known T-stage that also significantly validated the observation (Fig. 1e; *p* = 2 × 10^–5^; Kruskal–Wallis test. All three datasets consistently demonstrate increasing *SFRP4* expression with more advanced T-stage.

Expression of SFRP4 protein mirrored the mRNA result. Immunohistochemistry of SFRP4 was performed and staining was assessed semi-quantitatively on a scale 0–3 + . Increased SFRP4 protein expression was observed in T3 compared with T1/T2 tumours (Fig. 1f). SFRP4 was overexpressed in the majority of gastric cancers and absent in normal gastric tissue (Fig. 1g).

### Elevated SFRP4 mRNA expression predicts poor prognosis

**Fig. 2:**
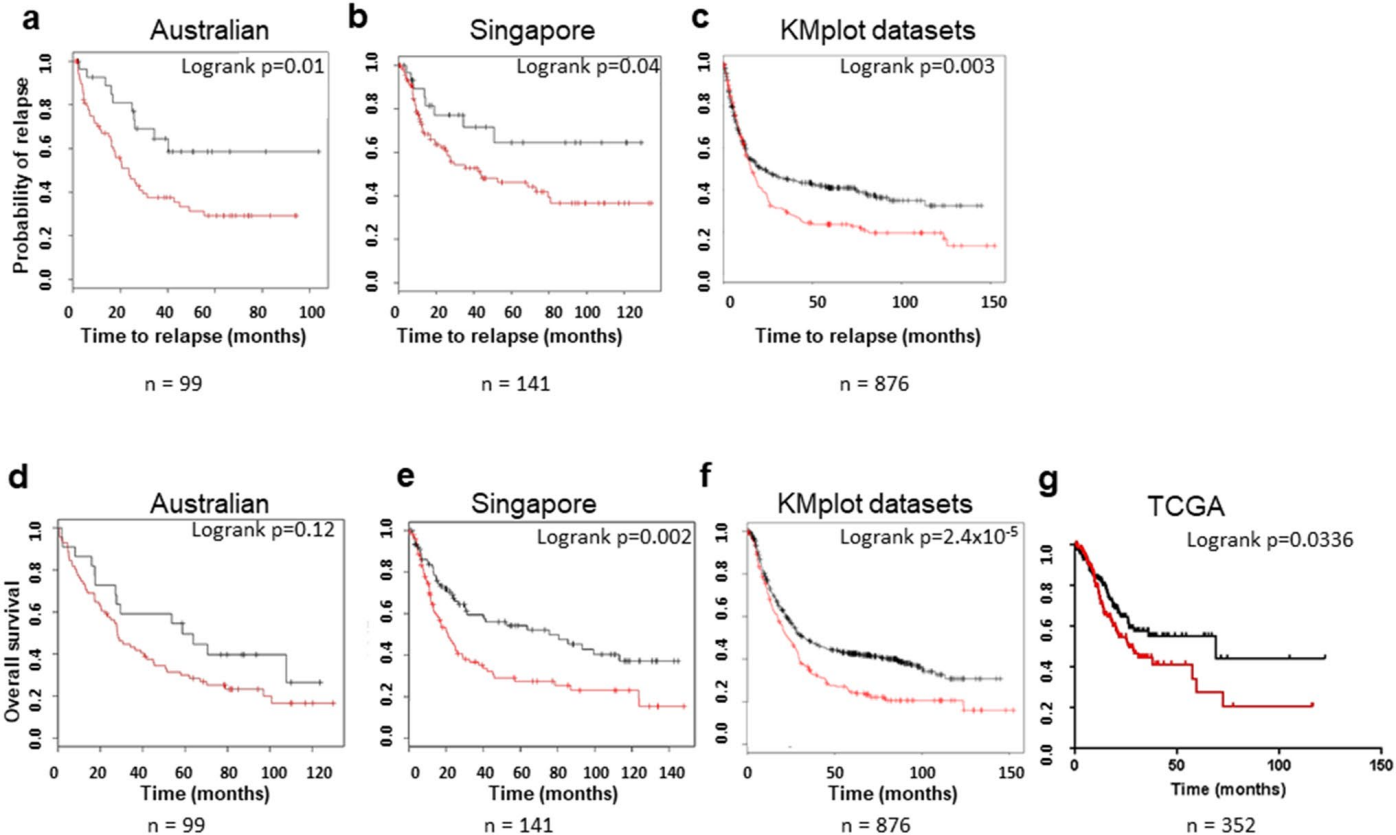
Effects of SFRP4 expression on relapse free (RFS) and overall survival (OS). Samples with available survival data were classified as SFRP4 high (red) or low (black) based on mRNA expression using the Barcode method [Australian (*n* = 99) and Singapore cohorts (*n* = 141)] or default settings of the kmplot interface (combined independent datasets (*n* = 876) or Survexpress for the TCGA STAD cohort (*n* = 352). **a-c** Kaplan–Meier curves were generated showing RFS. The results indicate that high SFRP4 expression levels were correlated with poor prognosis whilst patients harbouring tumours with low SFRP4 expression levels had a significantly lower risk of recurrence [*p* = 0.01 (Australian data set); *p* = 0.04 (Singapore data set); *p* = 0.003 (combined independent data set) log-rank test]. A similar analysis was performed using OS as an endpoint (**d**) Australia dataset *p* = 0.12 (**e**) Singapore dataset *p* = 0.002 (**f**) combined independent data set p = 2.4 × 10^–5^ (**g**) TCGA STAD dataset (*p* = 0.0336)

Affymetrix array-derived data were used to determine whether tumour *SFRP4* mRNA expression levels could reliably predict prognosis. The Barcode method [13] of analysis which uses a binary representation of expression data was used to classify samples as having high or low *SFRP4* mRNA expression. Relapse-free survival (RFS) indicated that high *SFRP4* mRNA expression levels were correlated with poor prognosis and patients with low *SFRP4* expression levels had a significantly lower risk of recurrence in both the Australian (*p* = 0.01; Fig. 2a) and Singapore (*p* = 0.04; Fig. 2b) datasets. These findings were also validated using the gastric cohorts in the KMplot database (*p* = 0.003; Fig. 2c) [14]. Survival data were limited for the TCGA dataset with no RFS data available.

A similar analysis with overall survival as an endpoint indicates that high SFRP4 expression correlates with poor survival, irrespective of T-stage in all four cohorts studied (Australian *p* = 0.12 Fig. 2d; Singapore *p* = 0.002 Fig. 2e; KMplot gastric datasets *p* = 2.4 × 10^–5^ Fig. 2f and TCGA STAD datasets *p* = 0.0336 Fig. 2g).

### SFRP4 has a functional role in invasion

**Fig. 3:**
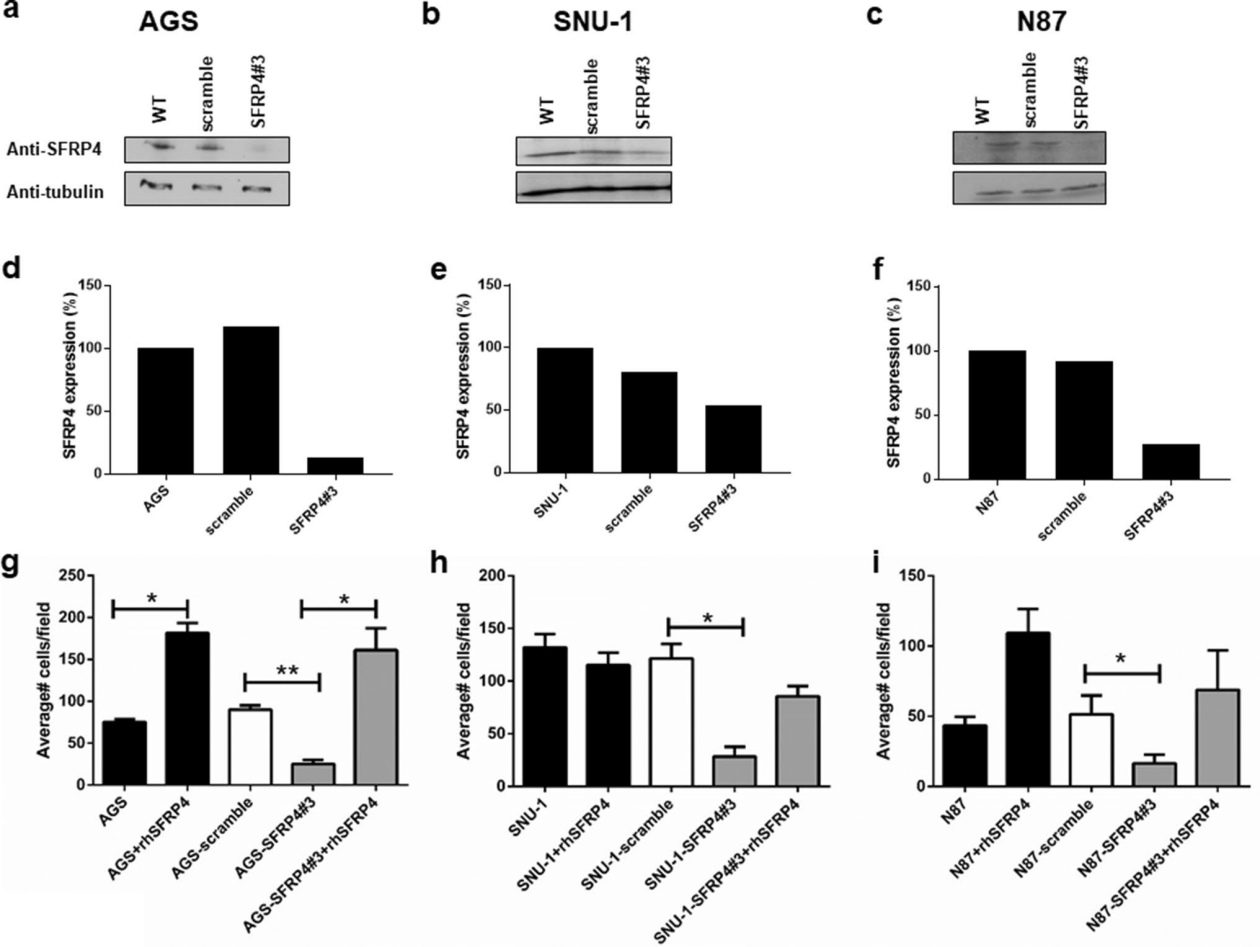
shRNA knockdown of gastric cancer cell lines. shRNA based lentiviral constructs were used to knockdown SFRP4 expression in (**a**) AGS, **b** SNU-1 and **(c)** NCI-N87 gastric cell lines using the SFRP4#3 construct and validated by Western blot. **d-f** Quantitation of knockdown was determined using Image J (**g-i**) Invasion assays were performed using WT cells, WT cells + scramble and WT cells + SFRP4 lentivirus. Reduction of SFRP4 expression resulted in a significant reduction in invasive capabilities (compared to scramble controls) in all cell lines. Pre-incubation of the cells with 20 nM recombinant human SFRP4 was able to restore the invasive ability of the knockdown cells to wild-type levels and, in the case of AGS and NCI-N87 cell lines was able to enhance the invasive capability of WT cells. Data show means ± SD. of at least three independent experiments. *p < 0.05, **p < 0.01

In view of the association with T stage, we wanted to examine the role of SFRP4 in the mechanism of invasion. shRNA was used to knockdown *SFRP4* expression in GC cell lines AGS, SNU-1 and NCI-N87. Western blot was used to confirm knockdown of SFRP4 expression at the protein level (Fig. S2A–C) and quantitation of knockdown is shown in Fig. S2D–F). The best overall knockdown was observed using construct #3 (Fig. 3a–f) which was then used for subsequent experiments.

Potential confounding effects of *SFRP4* knockdown on proliferation (Figs. S2G–) and apoptosis (Figs. S2I–L; Fig. S3A) were excluded.

A critical requirement for tumour invasion is the ability of the cancer cells to penetrate the extracellular matrix and ultimately invade into adjacent structures. We utilised invasion assays to quantitate the ability of GC cells to invade through matrigel in vitro*.* AGS cells with reduced *SFRP4* expression (AGS-SFRP4#3) exhibited a significant (72%) reduction in invasion compared to the non-targeting control (Fig. 3g; AGS-scramble; *p* = 0.0005; *t* test). Pre-incubation of knockdown cells with recombinant human-SFRP4 restored the invasion capacity of the cells to invade back to basal levels in a dose-dependent manner (Fig.S3B). The addition of 20 nM recombinant SFRP4 protein to wild-type cells also significantly enhanced their ability to invade over baseline Fig. 3g (*p* = 0.02; *t* test). This suggests the secreted form of SFRP4 can help drive invasion. Similar results were obtained with SNU-1 (*p* = 0.05) and NCI-N87 (*p* = 0.04;* t* test) cells, which also exhibited significant reduction (77% and 68% respectively) of invasive ability after SFRP4 knockdown (Figs.3h, i, respectively). Representative images of invasion for all cell lines tested are shown in Fig. S3C.

Migration assays showed that reduced SFRP4 levels inhibited the migration of AGS cells (*p *= 0.007; Fig. S3D). This assay was only performed for the AGS cell line as the other cell lines do not grow in a uniform monolayer, which is required for this assay.

Taken together, the validation and functional data described above suggest a functional role for secreted SFRP4 in the invasion of GC. To further explore this gene expression data from the Australian and TCGA cohorts was interrogated to identify genes most correlated with *SFRP4* (447 and 64 genes, respectively; Table S2 and Fig. S4A). Genes common to both datasets (*n* = 53; Table S2 and Fig. S4A) were further analysed using the Reactome Database [18]. Five of the top 8 enriched pathways are related to extracellular matrix organisation, composition and degradation. (Fig. S4B).

Given that disruption of the extracellular matrix promotes epithelial to mesenchymal transition (EMT) and ultimately invasion the Australian cohort was used to correlate *SFRP4* expression to that of key genes involved in EMT. *SFRP4* was found to be significantly positively correlated to *SNAI1*, *SLUG*, *VIM*, *ZEB1*, *ZEB2* and *TWIST1* and negatively correlated to *CDH1* (Fig. S5). These findings suggest that EMT may be a large component of the mechanism of action of SFRP4.

### Utility of SFRP4 as a biomarker of recurrence

Based on the finding that the secreted form of SFRP4 has biological relevance for GC we investigated the possibility that serum or plasma SFRP4 levels could be used as a diagnostic test for patients with GC or as a prognostic biomarker. SFRP4 ELISA showed a high patient-to-patient variation of both non-cancer volunteers and GC patients with no overall difference between the two groups (data not shown) suggesting that baseline or preoperative blood SFRP4 levels are not a suitable for diagnosis of GC.

The role of SFRP4 in invasion and potentially metastasis suggested a change in plasma levels within an individual may be useful in predicting patient recurrence after curative resection. We sought to determine whether SFRP4 levels changed after curative resection when compared to an individuals baseline level to test the change in SFRP4 level as a biomarker of tumour recurrence. We utilised a unique cohort of patients who had undergone curative resection and were followed for up to 10 years (mean 7.3 years in non-recurrence cases) with serial blood samples.

The pilot phase analysed 11 patients with recurrent disease from our Australian cohort and measured SFRP4 levels in plasma: pre-operatively (baseline-levels); post-operatively (first blood drawn post-surgery); pre-recurrence (blood drawn before clinical diagnosis of recurrence) and; post-recurrence (first blood drawn after clinical diagnosis of recurrence). The control group comprised 13 patients, matched for stage, treatment, age and gender and who had undergone curative gastric resection with no documented recurrence. For the control group, plasma collected: pre-operatively (baseline-levels); post-operatively (first blood drawn post-surgery) and; a time point at least 36 months post operatively was analysed. This time point was selected as most GC patients who recur do so within 36 months of surgery. Patient selection criteria (Fig. S1) and clinical characteristics (Table 1) were matched in both groups.

**Fig. 4:**
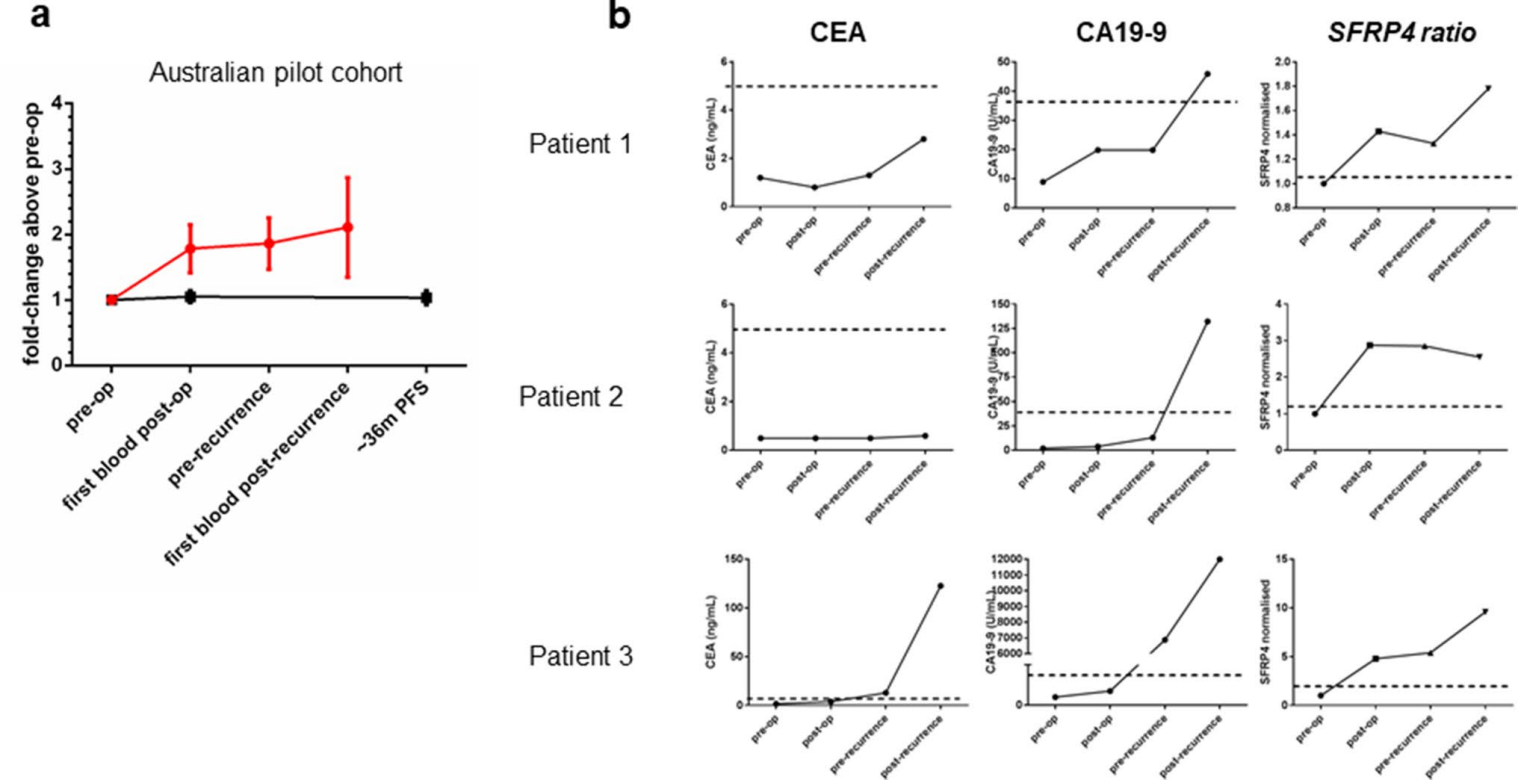
Secreted SFRP4 as a biomarker for gastric cancer. **a** Plasma samples collected over a period of 36 months from a pilot cohort of clinically matched Australian GC patients, 11 of whom ultimately recurred (red line) and 13 who did not recur (black line) were sampled using a commercial ELISA based assay. For each patient, all results were normalised to that of their pre-operative blood sample (*SFRP4 ratio*). **b** The ability of *SFRP4 ratio* to predict recurrence was tested in a series of patient samples collected from patients who have previously developed recurrent disease. For each patient CEA, CA19-9 and *SFRP4 ratio* levels were determined and compared using plasma collected pre-operatively, the first post-operative blood, pre documented recurrence (pre-recurrence) and following clinically confirmed recurrence (post-recurrence). Dotted lines represent the clinically utilised cut-offs for each test (CEA; 5 ng/mL and CA19-9; 35U/mL). For *SFRP4 ratio* the pre-determined cut off ratio of 1.2 was used. CEA yielded various results and was only able to predict recurrence in a small percentage of cases. CA19-9 only showed positive results after recurrence was detected clinically. These data negate the use of both these tests as a biomarker of GC. *SFRP4 ratio*s hows the promising ability to predict recurrence soon after curative resection

Plasma SFRP4-levels were determined for all samples using ELISA and were measured as a ratio against the individual patient’s baseline level, to control for individual variation. (Fig. 4a). SFRP4 ratio was defined as the ratio of the first post-operative blood SFRP4 level and the baseline SFRP4 level for individual patients. The data indicate that SFRP4 ratios remained constant for patients who did not develop a recurrence of disease, whilst there was an early and sustained increase over baseline of SFRP4 plasma ratios in patients who ultimately recurred (Fig. 4a). There was a clear increase in circulating SFRP4 levels in patients who develop cancer recurrence that occurs very early after curative resection and was maintained, in some cases for years, before the clinical diagnosis of recurrence (Fig. S6). We propose this novel finding could be exploited as a clinical biomarker of recurrence that would facilitate triage to more aggressive therapy or surveillance in the high-risk group.

### Comparison of SFRP4 ratio and existing blood-based biomarkers

There are no established prognostic or surveillance biomarkers recommended for GC. We compared *SFRP4 ratio* with existing clinical biomarkers for gastrointestinal cancer and upper gastrointestinal cancer, CEA and CA19-9 respectively, to evaluate their ability to pre-empt and detect recurrence. Pre-operative, post-operative, pre-recurrence and post-recurrence bloods from GC patients were analysed using the standard CEA and CA19-9 testing methods performed by the Peter MacCallum Cancer Centre Pathology service. Figure 4a shows representative data for 3 patients. CEA and CA19-9 show limited ability to detect existing recurrent disease prior to the clinical diagnosis of the recurrence, which provides no clinical advantage. *SFRP4 ratio* is the only marker elevated before recurrence, often months or years before the recurrence is clinically diagnosed.

### SFRP4 ratio and AJCC combination is the most accurate predictor of recurrence (PredictR)

Given the superior ability of *SFRP4* ratio to predict recurrence in comparison to existing biomarkers we then sought to determine whether this was independent of currently accepted clinical prognosticators, namely pathological TNM stage. Logistic regression was used to assess the role of each of 5 different models: (i) T-stage alone; (ii ) N-stage alone; (iii) *SFRP4 ratio* alone; (iv) pathological AJCC stage (7th edition) alone and; (v) *SFRP4 ratio* and pathological AJCC (7th edition) in combination using a validation cohort of 67 clinically matched GC patients from Australia which included the pilot group (Clinical data in Table 1).

**Fig. 5:**
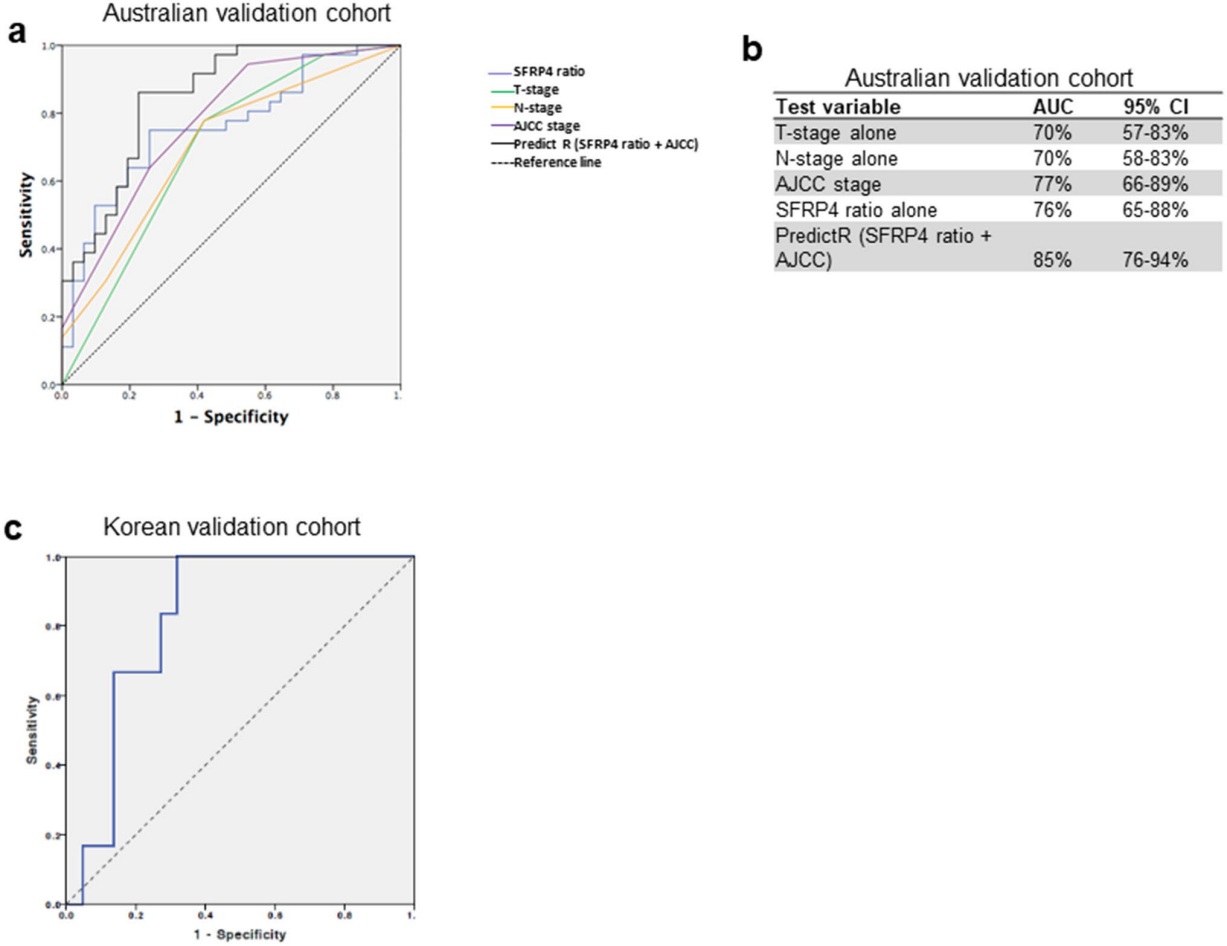
Development of an a test to predict recurrence of gastric cancer post resection (PredictR) (**a**) ROC and Logistic regression were used to determine whether *SFRP4 ratio* was predictive of recurrence independent of T-stage, N-stage and AJCC stage using an extended validation cohort of 67 Australian GC patients (36 recurrence and 31 non-recurrence). *SFRP4 ratio* and AJCC stage alone were similar in predictive accuracy when used independently, however when combined (PredictR) their accuracy was significantly improved. Odds ratio of recurrence using SFRP4 ratio alone at a cutoff of 1.21 was 7.2 (95% CI 2.5–20.6; *p* < 0.001) **b** Area under ROC of the different groups showing highest accuracy in the *PredictR* (SFRP4ratio/AJCC) combination using a logistic regression model with 95% CI (p-value for the difference with AJCC alone = 0.044) **c** This was further validated in a second independent cohort of 28 Stage III Korean patients (Korean validation cohort) 6 recurrence and 22 non-recurrence) and found an accuracy (AUC) of 83% using *SFRP4 ratio*. Odds ratio of recurrence with *SFRP4 ratio* at a cutoff of 1.21 was 16 (95% CI 1.5–171.2;*p* value = 0.002)

Models of prediction were evaluated using an ROC analysis (Figs.5a, b). The *SFRP4 ratio*/AJCC combination was termed PredictR and had an AUC of 85% (95%CI76–94%) for prediction of recurrent disease whereas pathological AJCC (the current preferred predictor of recurrence) was 77% (95%:CI 66–89%) and the p-value of this difference was 0.044. It is notable that *SFRP4 ratio alone *had an independent AUC of 76% (95% CI65–88%) and odds ratio 7.12 (95% CI2.5–20.6; *p *< 0.001) that the combination with pathological TNM (AJCC) was additive in the prediction accuracy.

This result was further validated using a prospectively collected series from South Korea. Based on the excellent prognosis of these patients, we selected cases with similar clinical profiles to our Australian cohort and we used 27 South Korean AJCC stage III cases. The ROC curve (Fig. 5c) reveals *PredictR* has an independent AUC of 83% (95% CI:63–98%) for prediction of recurrence in these patients. We performed logistic regression to determine if *SFRP4 ratio* alone at a threshold value of 1.2 was predictive of recurrence in this cohort and found an Odds Ratio of 16 (95% CI1.5–171.2; *p* = 0.022) independent of stage corresponding to a positive predictive probability of recurrence using *SFRP4 ratio* alone of 84%.

## Discussion

We have previously identified *SFRP4* as consistently overexpressed in all GC subtypes when compared to corresponding normal tissue [7]. Other studies have reported similar findings in a variety of solid cancers [12, 20–22]. Epigenetic downregulation of secreted frizzled-related proteins and *SFRP4* by promoter methylation has been described in some cancer types [23, 24] however in GC this seems to be restricted to the *SFRP2* gene [25]. Here we show that elevated tumour *SFRP4* expression is associated with poor prognosis in GC which was also recently reported by others [26]. These authors used an in silico based approach to show that overexpression of SFRP4 is correlated with poor survival in glioma, colorectal, clear cell kidney, liver, head/neck SCC and bladder cancers but not breast, lung, pancreatic, ovarian or oesophageal cancer, suggesting disparate roles for this protein in different tumour contexts [26].

Here, we used multiple clinical genomic cohorts of GC to validate the finding that *SFRP4* is over-expressed in more invasive cancers. Invasion is an important hallmark of malignancy [5, 6]. Our analysis was found to significantly predict the risk of recurrence of patients after curative resection of their GC. *SFRP4* tumour expression was previously reported as part of a 6-gene signature which was able to predict recurrence of GC [27]. Further, the prognostic value of *SFRP4* overexpression in resectable GC was recently validated in archived samples from the CLASSIC trial cohort [28]. We showed elevated SFRP4 was significantly associated with poor overall survival in the Singapore, KMplot and TCGA cohorts. A similar trend was also seen in the Australian cohort but this was not significant most likely due to the small sample size.

Despite being well known for their role as WNT modulators and tumour suppressors there is emerging evidence that suggests the SFRPs may have alternate functions related to the promotion of carcinogensis in a context and tumour type-dependent manner [29]. Indeed a pan-cancer, in silico study [26] suggests that despite their proposed roles as Wnt antagonists SFRP2 and SFRP4 may promote processes such as cellular invasion and metastasis. This is consistent with our findings which show that SFRP4 expression is correlated with T-stage. We observed that genes correlated with SFRP4 are functionally enriched for signalling and extracellular matrix pathways both of which are disrupted during EMT, a cellular de-differentiation process by which epithelial cells lose cell adhesion capabilities and gain invasive properties [30].

We find that reducing SFRP4 levels using shRNA resulted in the reduced invasion of different subtypes of gastric cancers in vitro. This novel finding introduces a unique variation to the treatment of cancer since we have found inhibiting *SFRP4*, while not oncocidal, may be considered oncostatic (limiting invasion and potentially metastasis).

A second novel finding describes an assay which uses SFRP4 plasma levels in two blood samples of humans with GC. The first is taken immediately before surgery for curative resection and the second at approximately 1 month following surgery. The ratio of these values (*SFRP4 ratio*) allows for prediction of recurrence many months or years into the future with an early and sustained increase in SFRP4 levels observed following curative surgery in patients whose cancer ultimately recurred. The only other use of *SFRP4* as a biomarker was in the context of Type2 diabetes mellitus [31]. We considered the potential confounding of Type2 diabetes mellitus in our study participants and found an equivalent distribution of patients in our cohort (Table1).

There are currently no effective non-invasive prognostic biomarkers for the early detection of GC or its recurrence following curative resection. Pepsinogen I/II ratio [32] has been used as a screening test in some countries but is not useful as a surveillance tool. Currently, there are no reliable circulating biomarkers for monitoring treatment of GC. Clinicians variably use CEA (carcinoembryonic antigen), CA19-9 and CA72-4 as serum markers. These assays were used mainly as diagnostic biomarkers rather than markers of prognosis. CA72-4 was reported with significant specificity (97%) for GC [33], but because of poor sensitivity (47%) is not used in population-based screening or for disease follow-up. Our *SFRP4 ratio* biomarker outperforms current serum-based tests in pre-empting recurrence many months prior to clinical detection of recurrence. Whilst significant when used alone, the predictive value of SFRP4 ratio is further improved with the addition AJCC staging data obtained at the time of surgical resection. We believe this is a major step forward in providing more precise information to patients and potentially changing their management decisions. This observation however needs to be replicated in prospective trials. A number of potential biomarkers for GC, based on genetic and epigenetic changes, have been described. However, none of these have translated into clinical use, again due to lack of sensitivity and/or specificity (reviewed in [34, 35]).

One limitation of this study reflects the current clinical care of GC patients after curative resection. Namely, the guidelines for postoperative management of GC recommend clinical follow-up but no active radiological follow-up [36] because there is currently limited evidence suggesting that early detection of recurrence impacts patient survival. As a result of current clinical practice, patients within this cohort were only investigated for recurrence after clinical suspicion. It is conceivable that recurrence in our *PredictR *positive patients may have been detected earlier if regular surveillance was the standard of care. We believe the results presented in this study warrant further clinical trial to validate the efficacy of in a prospective cohort and we believe may lead to practice change in GC management by allowing us to identify patients at risk of recurrence very early and allow targeted therapy or more intensive surveillance of that high-risk group.

In conclusion, we have shown that *SFRP4* is over-expressed in the majority of gastric cancers and its high expression leads to a poor outcome. We report for the first time that *SFRP4* functions in the cellular invasion of GC and this is the reason for the poor prognosis and that inhibition of SFRP4 leads to abrogation of invasion in vitro. This study also found that SFRP4 levels in patients after curative resection of gastric cancer predicts GC recurrence at an early stage. We hypothesise that resection of the primary tumour triggers the establishment of a permissive environment for pre-seeded micrometastases to begin to invade, prior to the clinical detection, and that this evidenced by an early and sustained increase in serum SFRP4 levels in patients who ultimately recur. Our *PredictR* assay may allow triage of patients to a high-risk group warranting more aggressive therapy and increased surveillance. This is a step towards precision medicine given *PredictR* may be used in conjunction with a companion therapeutic that could target SFRP4 inhibition. These will need to be tested in future clinical trials to determine whether this strategy leads to improved clinical outcomes.

## Supplementary Information

Below is the link to the electronic supplementary material.

Supplementary file1 (PDF 1388 KB)
