# Supplementary material for: SFRP4 drives invasion in gastric cancer and is an early predictor of recurrence
Source: Gastric Cancer. 2020 Dec 4;24(3):589–601. doi: 10.1007/s10120-020-01143-8 (PMC8064978; doi:10.1007/s10120-020-01143-8)
Supplement: Supplementary file 1 — Supplementary file1 (PDF 1388 KB) [file 10120_2020_1143_MOESM1_ESM.pdf]

SUPPLEMENTARY FILES

Journal: Gastric Cancer

SFRP4 drives invasion in gastric cancer and is an early predictor of recurrence

Rita A. Busuttil[1,2,3], Joshy George[4], Colin M. House[1], Stephen Lade[5], Catherine Mitchell[5], Natasha S. Di Costanzo[1], Sharon Pattison[6], Yu-Kuan Huang[1,2,3], Patrick Tan[7,8], Jae-Ho Cheong[9,10], Sun Young Rha[11] & Alex Boussioutas[1,2,3]

[1] Upper Gastrointestinal Translational Research Laboratory, Peter MacCallum Cancer Centre, Parkville, Victoria, Australia
[2] Sir Peter MacCallum Department of Oncology, The University of Melbourne, Parkville, Victoria, Australia
[3] Department of Medicine, The University of Melbourne, Parkville, Victoria, Australia
[4] Computational Sciences, Jackson Laboratory for Genomic Medicine, Farmington, Connecticut USA
[5] Department of Pathology, Peter MacCallum Cancer Centre, East Melbourne, Victoria, Australia
[6] Department of Medicine, University of Otago, Dunedin, New Zealand
[7] Genome Institute of Singapore, Singapore.
[8] Cancer Science Institute of Singapore, Yong Loo Lin School of Medicine, National University of Singapore, Singapore
[9] Department of Surgery, Yonsei University College of Medicine, Seoul, Republic of Korea
[10] Department of Biomedical Systems Informatics, Yonsei University College of Medicine, Seoul, Republic of Korea
[11] Division of Medical Oncology, Yonsei Cancer Center, Yonsei University College of Medicine, Seoul, Republic of Korea.

Corresponding author info: Professor Alex Boussioutas,Peter MacCallum Cancer Centre, 305 Grattan St, Parkville Telephone: +61 3 98559 7031, Fax: +61 3 8559 5489, Email: alex.boussioutas@petermac.org

Table S1: Clinical characteristics of Australian GC cases

Parameter	Discovery cohort<br>No.	Validation cohort<br>No.
Age (years)		
Male	64 (32-83)	65.4 (32-83)
Female	73.7 (47-85)	67 (33-86)
Gender		
Male	45	67
Female	20	32
Tumour location		
GOJ/Cardia	10	20
Greater curve-body	13	28
Lesser curve-body	19	29
Body unspecified	5	1
Antrum	14	20
Stomal	0	1
Chemo-radiotherapy		
Adjuvant		
No Adjuvant	34	40
Adjuvant	14	46
ND	15	13
Palliative		
No Palliative	25	21
Palliative	13	24
NA	12	0
ND	15	54
Pathology		
Diffuse	23	39
Intestinal	35	50
Mixed	6	10
Adenosquamous	1	0
Differentiation		
Well	4	3
Moderate	13	31
Moderate to poor	6	0
Poor	20	35
Undifferentiated	20	29
Other	2	1
T Stage		
T1	7	10
T2	14	25
T3	44	62
T4	0	2
AJCC Stage		
IA	3	5
IB	9	14
II	14	26
IIIA	22	23
IIIB	13	22
IV	3	8
Unspecified	1	1

NA; type of therapy not applicable, ND; data not available

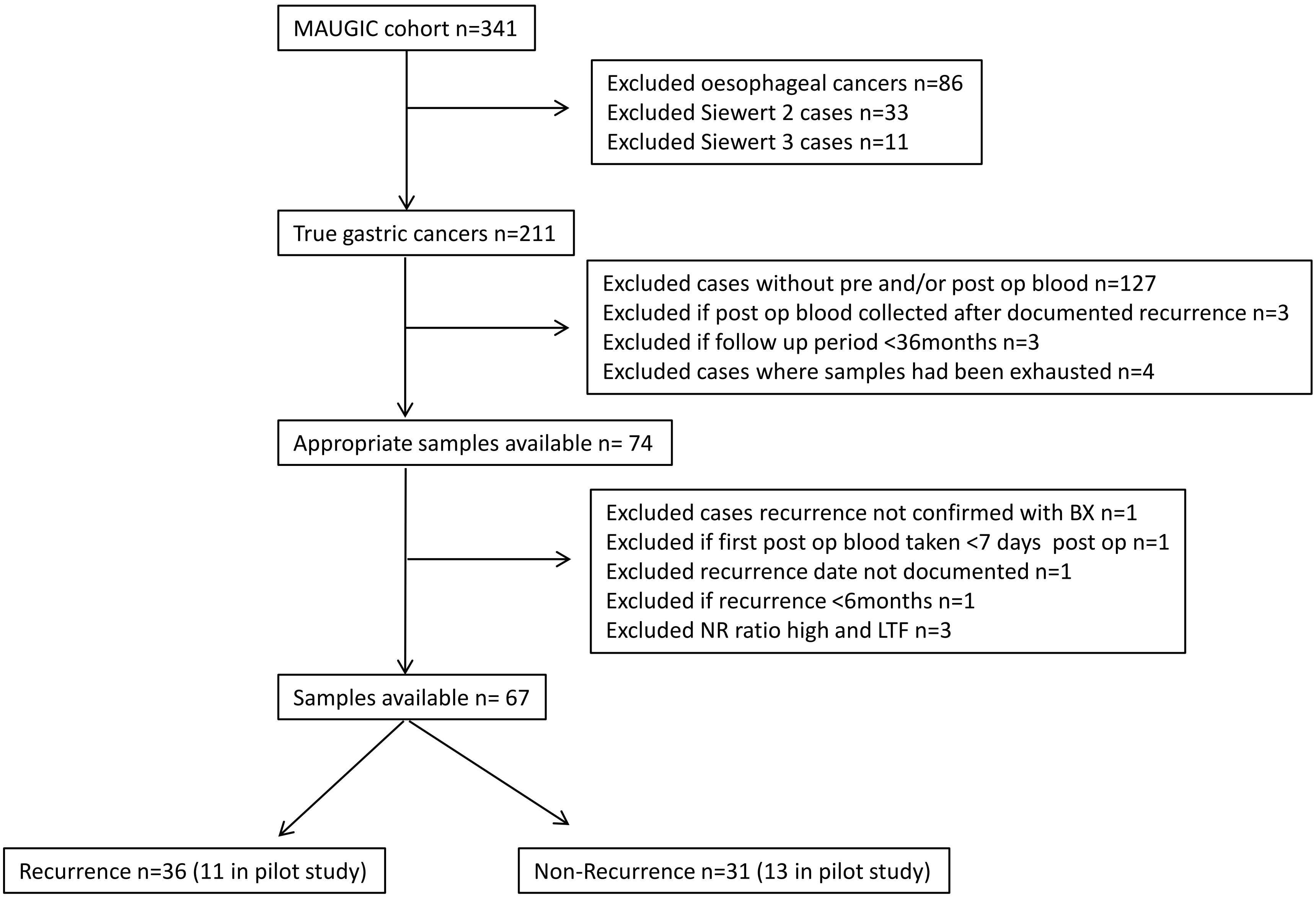

Figure S1. Consort diagram for the selection of gastric cancer plasma samples for SFRP4 ELISA.

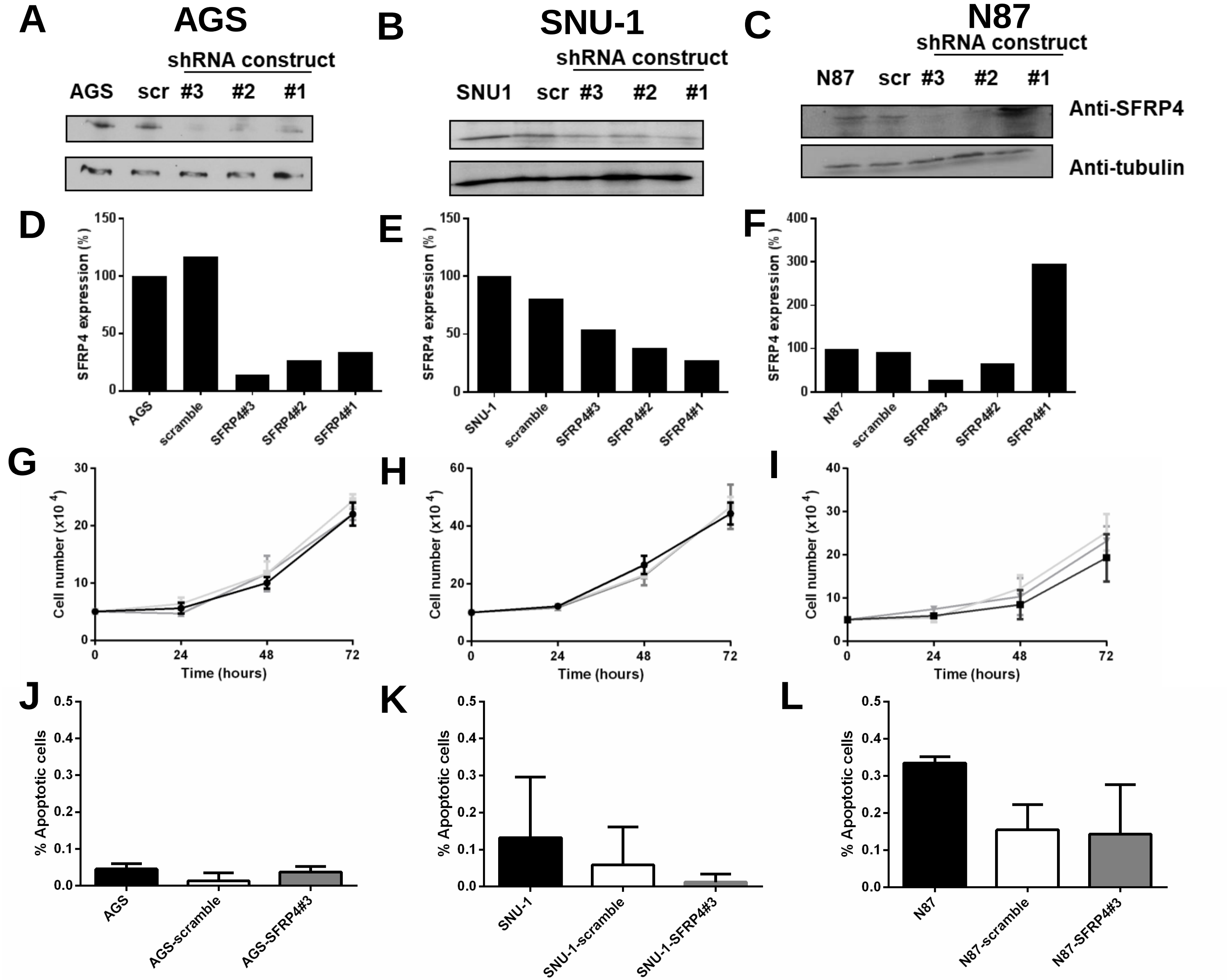

Figure S2. Effects of shRNA mediated knockdown of SFRP4. shRNA mediated knockdown of gastric cancer cell lines (A) AGS, (B) SNU-1 and (C) NCI-N87 was performed using the three available constructs targeting the SFRP4 gene (Supplementary Table S2) and a scramble control. Western blots were performed to confirm knockdown and the resulting immunoblots in (A-C) were quantitated by densitometric methods (D-F) using Image J. Overall construct #3 was the best performing and used for all subsequent experiments. Proliferation was determined by cell counts at 24, 48 and 72 hours (G-I) cell lines. No significant differences were observed in any of the cell lines. (J-L) Apoptosis was determined for the same cell lines using the Apoptaq kit. Representative images are shown in Fig. S3. Data are represented as mean ± SD.

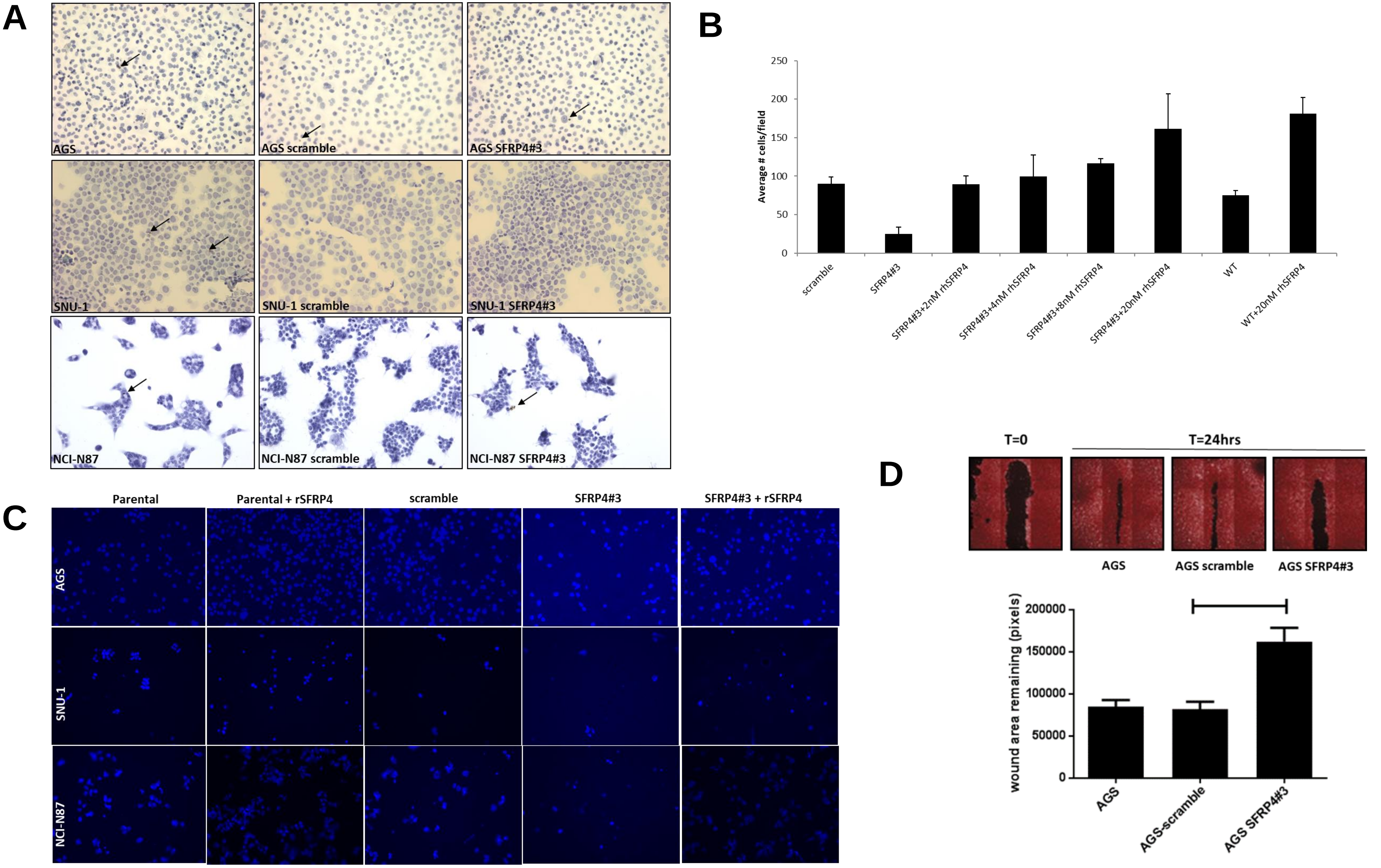

Figure S3. Outcomes of functional assays **(A)** representative images from apoptosis assays (arrows represent apoptotic cells), **(B)** the effect of the addition of recombinant human SFRP4 on the invasion of AGS cells. AGS WT or AGS-SFRP4 knockdown cells were pre-incubated with varying doses of rhSFRP4 before use in an invasion assay. Data are represented as mean ± SD ,**(C)** representative images of AGS, SNU-1 and NCI-N87 cells from invasion assays shown in Fig 3. **(D)** AGS wound healing assay. AGS WT, scramble and SFRP4 knockdown cells were seeded to confluency, scratched to wound and imaged at 0- and 24-hours.Wound healing was then quantitated *p=6.8x10^{-3}; t test. Data are represented as mean ± SD.

Table S2: Genes correlated with SFRP4

Australian dataset														TCGA dataset		Common genes
Gene	Pearson Score	Gene	Pearson Score	Gene	Pearson Score	Gene	Pearson Score	Gene	Pearson Score	Gene	Pearson Score	Gene	Pearson Score	Gene Symbol	Pearson Score	Gene Symbol
ACTA2	0.71	CNN1	0.60	FAM20C	0.72	ISM1	0.70	MRAS	0.76	PROS1	0.65	STON1	0.78	ADGRA2	0.61	ADGRA2
ACTN1	0.72	CNRIP1	0.65	FAM229B	0.62	ITGA1	0.60	MRC2	0.78	PRRX1	0.79	SULF1	0.76	AEBP1	0.67	AEBP1
ADAMTS2	0.62	COL10A1	0.73	FAP	0.81	ITGB5	0.68	MRGPRF	0.69	PRSS23	0.65	SVIL	0.67	ANTXR1	0.69	ANTXR1
ADARB1	0.64	COL12A1	0.61	FBLN1	0.68	ITGBL1	0.90	MRVI1	0.66	PTGFR	0.62	SYNC	0.74	ASPN	0.66	ASPN
ADGRA2	0.63	COL14A1	0.64	FBLN2	0.75	JAM3	0.73	MSC-AS1	0.71	PTGIS	0.71	SYNDIG1	0.60	AZIN2	0.63	BGN
ADGRD1	0.65	COL16A1	0.65	FBN1	0.84	JAZF1	0.60	MSRB3	0.77	PYGL	0.65	SYT11	0.68	BGN	0.7	BICC1
AEBP1	0.83	COL1A1	0.60	FBXL7	0.68	KANK2	0.63	MXRA7	0.76	QKI	0.69	TAGLN	0.72	BICC1	0.62	C1R
AKAP12	0.62	COL3A1	0.75	FBXO32	0.71	KATNAL1	0.65	MXRA8	0.80	RAB23	0.72	TBCEL	0.61	C1QTNF5	0.61	C1S
AKT3	0.73	COL5A1	0.72	FERMT2	0.74	KCNE4	0.64	MYH10	0.79	RAB31	0.81	TCEAL7	0.63	C1R	0.67	CDH11
AMOTL1	0.74	COL5A2	0.67	FEZ1	0.67	KCNJ8	0.63	MYL9	0.68	RAI14	0.68	TCF4	0.72	C1S	0.64	CMTM3
ANGPTL2	0.73	COL6A1	0.61	FGF7P3	0.63	KCTD10	0.68	MYLK	0.70	RARRES2	0.71	TENM4	0.70	CDH11	0.78	COL8A1
ANKRD10	0.61	COL6A2	0.78	FGFR1	0.72	KDELC1	0.67	NALCN	0.66	RASL12	0.61	TGFB1I1	0.71	CMTM3	0.7	CTHRC1
ANOS1	0.77	COL6A3	0.64	FIBIN	0.66	KIAA1755	0.65	NAP1L3	0.72	RASSF8	0.74	TGFB3	0.64	COL8A1	0.73	DACT1
ANTXR1	0.89	COL8A1	0.92	FKBP14	0.60	KIDINS220	0.63	NAV3	0.63	RASSF8-AS1	0.60	TGFBR1	0.66	CPZ	0.67	DOK5
ANXA6	0.66	COL8A2	0.85	FKBP7	0.73	KIF1B	0.64	NDN	0.72	RBFOX2	0.64	THBS2	0.87	CTHRC1	0.62	ECM2
AOC3	0.63	COLEC12	0.66	FLNA	0.66	KIRREL1	0.74	NEXN	0.67	RBMS1	0.66	THBS3	0.75	DACT1	0.66	EFEMP2
AP1S2	0.67	COMP	0.66	FMO1	0.60	KLC1	0.68	NID2	0.64	RBMS3	0.67	THBS4	0.73	DOK5	0.63	ELN
ARL10	0.73	COPS8	0.61	FN1	0.72	KLHL29	0.66	NISCH	0.61	RCBTB2	0.61	THY1	0.78	ECM2	0.61	FAM19A5
ARMCX1	0.68	COPZ2	0.80	FNDC1	0.82	LAMA4	0.68	NNMT	0.61	RECK	0.65	TIMP1	0.75	EFEMP2	0.6	FAP
ARMCX2	0.61	COX7A1	0.69	FOXP2	0.61	LAMB2	0.68	NOTCH2	0.71	RERG	0.67	TIMP2	0.83	ELN	0.68	FBN1
ASPN	0.84	CPXM2	0.78	FRMD6	0.72	LAMC1	0.65	NOX4	0.72	RFTN1	0.66	TIMP3	0.73	FAM19A5	0.62	FNDC1
ATL3	0.64	CRISPLD1	0.81	FSTL1	0.84	LARP6	0.64	NR2F1-AS1	0.62	RGL1	0.63	TMEM173	0.61	FAP	0.6	FSTL1
ATP10A	0.73	CRTC3	0.62	FTO	0.61	LATS2	0.64	NREP	0.75	RGMA	0.63	TMEM43	0.67	FBN1	0.63	GFPT2
ATP8B2	0.61	CRYAB	0.66	FUT11	0.71	LAYN	0.70	NRK	0.62	RHOQ	0.62	TMEM45A	0.62	FNDC1	0.71	GLIS2
ATXN1	0.63	CSGALNACT2	0.75	FXYD6	0.65	LBH	0.65	NRP2	0.70	RILPL1	0.64	TMEM47	0.65	FSTL1	0.61	GLT8D2
BACE1	0.61	CTHRC1	0.73	FZD1	0.73	LGALS1	0.72	NRXN3	0.67	RIMKLB	0.62	TNRC6C	0.60	GFPT2	0.6	IGFBP7
BGN	0.83	CTSK	0.79	GAL3ST4	0.64	LHFPL6	0.71	NTM	0.61	RNF144A	0.73	TNS1	0.71	GGT5	0.64	ISLR
BHLHE22	0.65	CYBRD1	0.60	GAS1	0.73	LINC01279	0.73	NUAK1	0.72	RNF146	0.60	TPM1	0.67	GLI1	0.7	ITGBL1
BICC1	0.82	CYP1B1	0.73	GAS7	0.74	LIX1L	0.71	OLFML1	0.62	RNF150	0.66	TPM2	0.70	GLIS2	0.64	KIAA1755
BMPR2	0.68	CYP2U1	0.63	GFPT2	0.71	LOC101929122	0.65	OLFML2B	0.74	RNF217	0.75	TRIO	0.66	GLT8D2	0.63	LOXL1
BNC2	0.75	DAB2	0.67	GJA1	0.62	LOX	0.63	OMD	0.67	ROBO1	0.67	TRPC1	0.71	HIC1	0.63	LRRC32
BNIP2	0.63	DACT1	0.75	GLI2	0.63	LOXL1	0.66	PALLD	0.74	ROR2	0.69	TRPS1	0.66	HTRA3	0.63	LTBP2
BOC	0.76	DCLK1	0.64	GLI3	0.72	LRCH2	0.61	PARD3B	0.62	RSPO3	0.68	TSHZ2	0.72	IGFBP7	0.63	LUM
C14orf132	0.61	DCLK2	0.61	GLIPR1	0.63	LRP12	0.61	PARVA	0.70	RTN1	0.62	TSHZ3	0.74	IGFBP7-AS1	0.62	MMP2
C1orf216	0.60	DCN	0.74	GLIS2	0.77	LRRC32	0.69	PBX3	0.63	RUFY3	0.62	TSPAN4	0.64	ISLR	0.77	MOXD1
C1orf54	0.63	DDR2	0.75	GLT8D2	0.79	LRRN4CL	0.65	PCOLCE	0.68	RUNX1T1	0.80	TSPAN9	0.65	ISLR2	0.62	MRC2
C1QTNF3	0.63	DENND5A	0.68	GNAL	0.63	LSAMP	0.63	PDE3A	0.64	S1PR3	0.64	TUB	0.62	ITGA11	0.62	MXRA8
C1R	0.78	DEPP1	0.68	GNB4	0.74	LTBP1	0.69	PDGFC	0.67	SAMD4A	0.61	TUBA1A	0.78	ITGBL1	0.76	NALCN
C1S	0.72	DIP2C	0.61	GPC6	0.63	LTBP2	0.76	PDGFRB	0.74	SCARF2	0.64	TUBB6	0.63	KIAA1755	0.65	NOX4
C3	0.62	DKK3	0.68	GPX8	0.75	LTBP3	0.66	PDGFRL	0.76	SCRG1	0.65	TWIST2	0.62	LOXL1	0.64	NREP
C4orf3	0.65	DNM3OS	0.70	GREM1	0.75	LUM	0.79	PDLIM2	0.62	SDC2	0.69	TWSG1	0.60	LRRC32	0.72	OLFML2B
C8orf88	0.60	DOCK11	0.67	GUCY1A3	0.74	MACF1	0.63	PDLIM3	0.75	SELENOM	0.69	UBE2E2	0.72	LTBP2	0.71	OMD
CALD1	0.82	DOK5	0.67	GUCY1B3	0.74	MAFB	0.66	PDLIM4	0.63	SERPINE2	0.66	UBE2Q2	0.63	LUM	0.71	PDGFRB
CALHM2	0.75	DPYSL3	0.85	GXYLT2	0.77	MAGI2-AS3	0.71	PDZRN3	0.69	SERPINF1	0.80	VAT1	0.64	LZTS1	0.61	PLXDC2
CALHM5	0.71	DSE	0.73	HDGFL3	0.76	MAN1C1	0.65	PEA15	0.77	SERPING1	0.75	VCAN	0.79	MMP2	0.63	SCARF2
CAMSAP2	0.63	DZIP1	0.69	HECTD2	0.68	MAP1A	0.68	PEAK1	0.62	SETBP1	0.61	VEGFC	0.61	MOXD1	0.74	SERPINF1
CAP2	0.64	ECM2	0.75	HEG1	0.75	MAP1B	0.66	PEG3	0.62	SFRP2	0.74	VGLL3	0.70	MRC2	0.63	SPARC
CARMN	0.63	EDNRA	0.69	HEYL	0.65	MAP3K20	0.66	PHLDA3	0.64	SGCD	0.67	VIM	0.68	MXRA8	0.71	THBS2
CAVIN1	0.79	EFEMP1	0.67	HMCN1	0.81	MAP3K3	0.67	PHLDB2	0.63	SGCE	0.68	VSTM4	0.66	NALCN	0.7	THY1
CAVIN3	0.61	EFEMP2	0.85	HOOK3	0.65	MAP4	0.65	PKD2	0.78	SH3PXD2A	0.63	WISP1	0.69	NOX4	0.74	TIMP2
CCDC80	0.78	EFS	0.65	HOXB2	0.62	MARVELD1	0.74	PLAGL1	0.62	SH3PXD2B	0.64	WWTR1	0.70	NREP	0.68	VCAN
CD109	0.61	EID1	0.62	HSD11B1	0.64	MDFIC	0.63	PLPPR4	0.62	SH3RF3	0.61	ZBTB20	0.60	OLFML2B	0.63	VEGFC
CDC42EP3	0.65	ELN	0.71	HSPB2	0.65	MEIS1	0.65	PLXDC1	0.60	SLC24A3	0.70	ZBTB4	0.61	OMD	0.61	ZNF521
CDH11	0.87	EMILIN1	0.72	HTRA1	0.62	MEOX2	0.65	PLXDC2	0.84	SLIT2	0.73	ZCCHC24	0.75	PDGFRB	0.66	
CDK14	0.63	EML1	0.69	IFFO1	0.63	MFAP2	0.67	PMP22	0.66	SMOC2	0.74	ZEB1	0.74	PLXDC2	0.67	
CDO1	0.61	ENOX1	0.61	IGFBP4	0.66	MFAP5	0.66	PODN	0.67	SOCS5	0.74	ZFHX3	0.64	SCARF2	0.66	
CDON	0.61	EPDR1	0.63	IGFBP6	0.67	MGP	0.77	PRDM2	0.63	SPARC	0.83	ZFPM2	0.74	SERPINF1	0.65	
CHRDL1	0.60	EPHA3	0.66	IGFBP7	0.72	MIR100HG	0.74	PRDM6	0.65	SPARCL1	0.64	ZNF25	0.68	SPARC	0.63	
CLEC11A	0.73	EVC	0.83	IL17RD	0.62	MIR99AHG	0.67	PRELP	0.75	SPART	0.65	ZNF423	0.73	THBS2	0.7	
CLIC4	0.74	EVC2	0.61	IL1R1	0.60	MMP2	0.74	PRICKLE1	0.69	SPIN1	0.65	ZNF436	0.73	THY1	0.6	
CLIP3	0.62	FAM198A	0.62	INHBA	0.73	MN1	0.76	PRICKLE2	0.62	SPOCK1	0.85	ZNF521	0.66	TIMP2	0.65	
CLIP4	0.63	FAM198B	0.75	INMT	0.66	MORF4L1	0.65	PRKD1	0.76	SPON1	0.66	ZNF532	0.63	VCAN	0.61	
CLMP	0.66	FAM19A5	0.69	IRF2BPL	0.67	MOXD1	0.82	PRKG1	0.64	SSPN	0.74	ZNF667-AS1	0.61	VEGFC	0.6	
CMTM3	0.75	FAM208A	0.66	ISLR	0.87	MPDZ	0.74	PRNP	0.61	ST6GALNAC5	0.63			ZNF521	0.6	

A

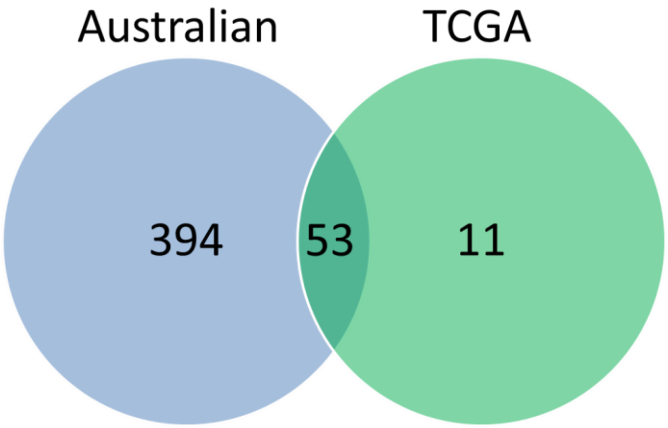

B

Pathway name	Entities				Reactions	
	found	ratio	p-value	FDR	found	ratio
Extracellular matrix organisation	14/329	0.023	1.07E-09	3.63e-07	77/318	0.025
Regulation of Insulin-like Growth Factor (IGF) transport and uptake by Insulin-like Growth Factor Binding Proteins (IGFBPs)	9/127	0.009	1.85e-08	3.14e-06	9/14	0.001
Elastic fibre formation	6/46	0.003	1.49e-06	1.68-05	12/17	0.001
Post-translational protei phosphorylation	7/109	0.008	1.48e-06	1.11e-04	1/1	8.01e-05
Molecules associated with elastic fibres	5/38	0.003	1.64e-06	1.11e-04	5/10	8.01e-04
ECM proteoglycans	5/79	0.005	5.42e-05	0.003	5/23	0.002
Diseases associated with glycosaminoglycan metabolism	4/55	0.004	1.86e-04	0.009	12/29	0.002
Degradation of extracellular matrix	5/148	0.01	9.55e-04	0.026	34/105	0.008

Figure S4. Genes and pathways correlated with SFRP4. (A) Genes commonly expressed in the Australian and TCGA cohorts were identified (Pearson correlation >0.6). **(B)** Top 8 functional pathways found to be enriched after further analysis of these genes using Reactome.

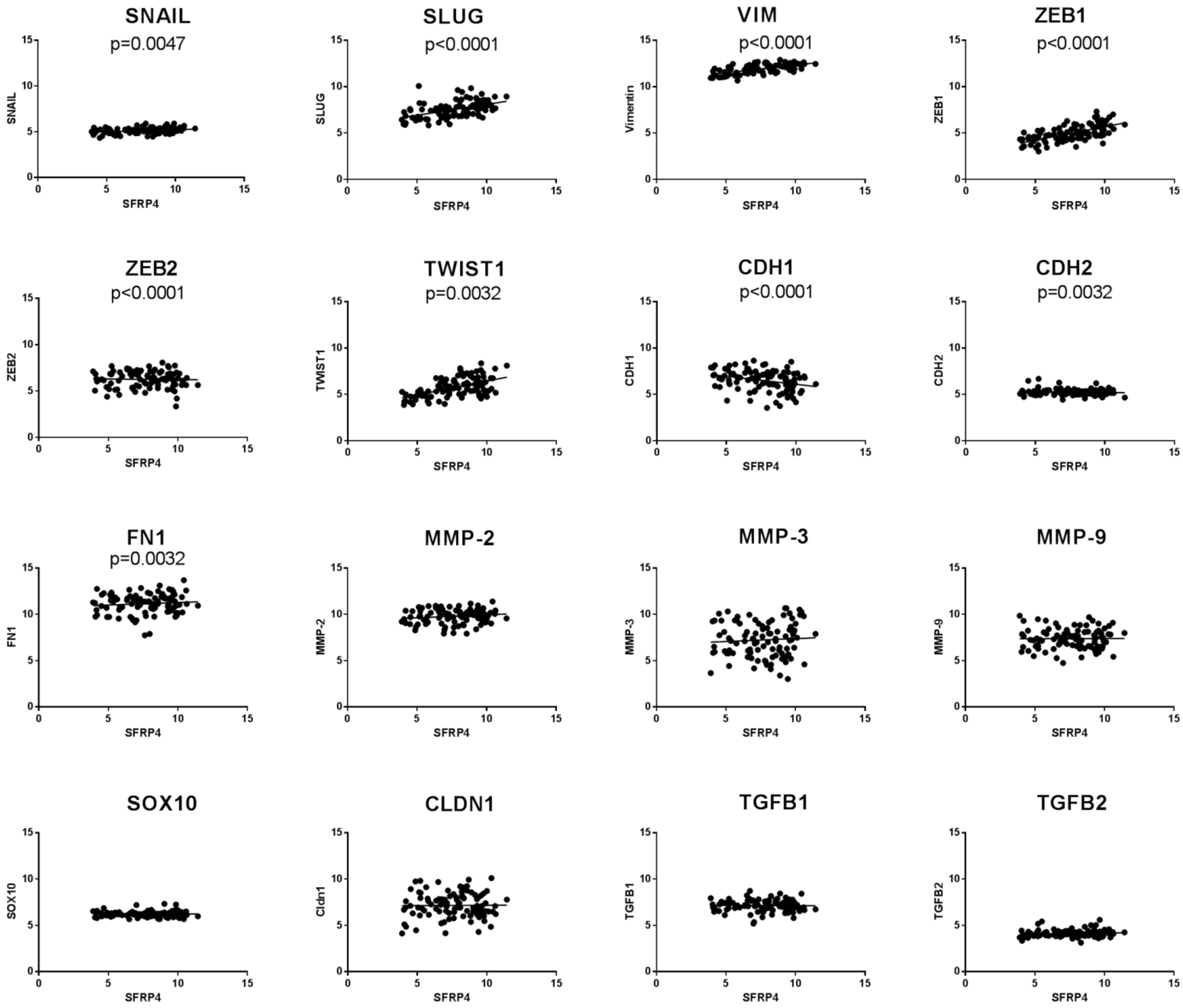

Figure S5. Correlation of SFRP4 expression with EMT related genes. Affymetrix U133 plus 2 mRNA data from the Australian dataset was used to correlate expression of SFRP4 with key EMT related genes

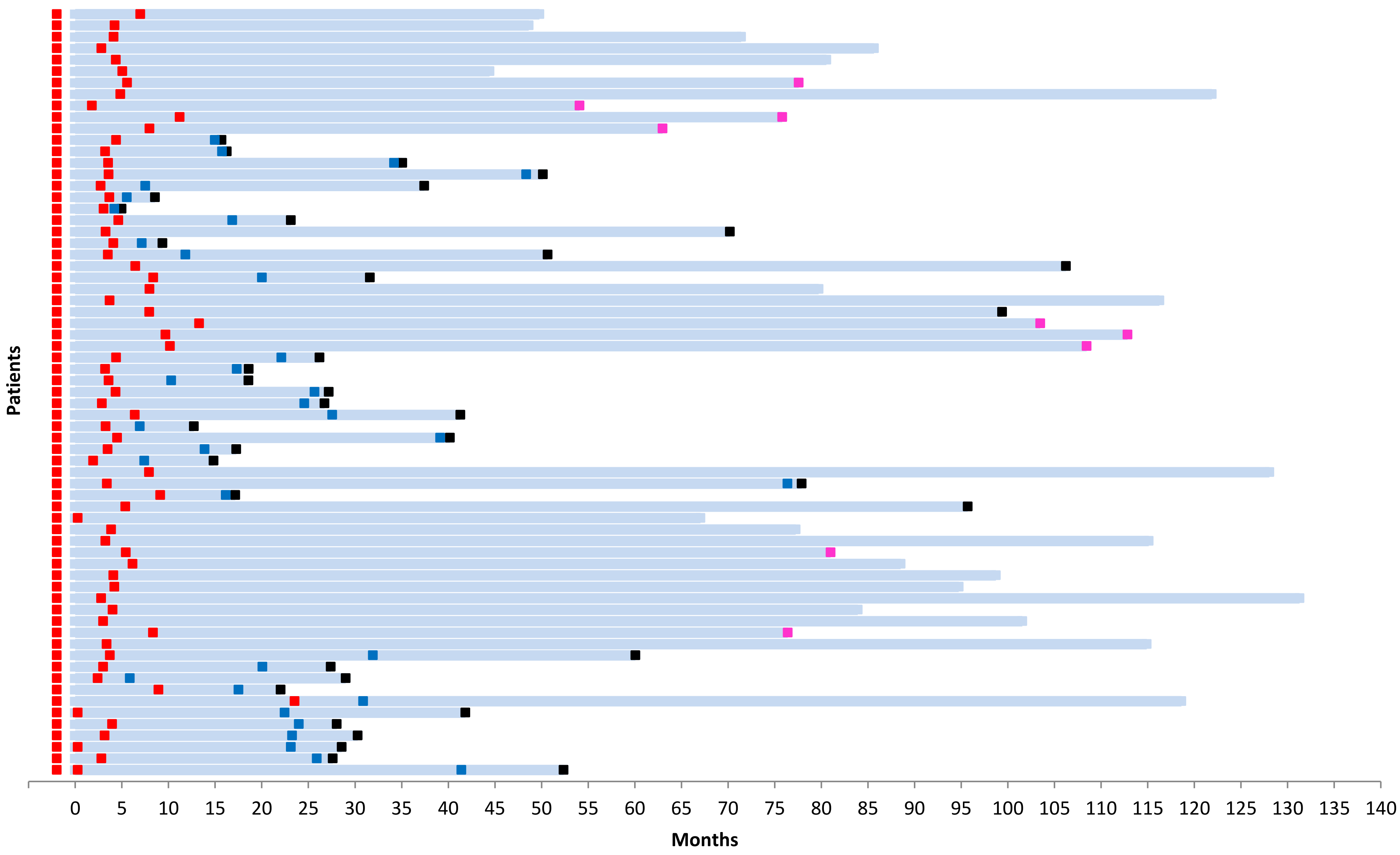

Figure S6. Swimmer plots outlining timelines for sample collection, recurrence and patient prognosis in the Australian cohort (n= 67). Zero months indicates the date of surgery. Key: Red= plasma sample collections; blue= time of clinically documented recurrence; black = overall survival; pink =lost to follow up
